# Tumour-infiltrating regulatory T cell density before neoadjuvant chemoradiotherapy for rectal cancer does not predict treatment response

**DOI:** 10.18632/oncotarget.15048

**Published:** 2017-02-03

**Authors:** Melanie J. McCoy, Chris Hemmings, Chidozie C. Anyaegbu, Stephanie J. Austin, Tracey F. Lee-Pullen, Timothy J. Miller, Max K. Bulsara, Nikolajs Zeps, Anna K. Nowak, Richard A. Lake, Cameron F. Platell

**Affiliations:** ^1^ Colorectal Research Unit, St John of God Subiaco Hospital, Subiaco, WA, 6008, Australia; ^2^ Department of Anatomic Pathology, St John of God Pathology, Wembley, WA, 6014, Australia; ^3^ School of Surgery, University of Western Australia, Crawley, WA, 6009, Australia; ^4^ School of Medicine and Pharmacology, University of Western Australia, Crawley, WA, 6009, Australia; ^5^ Institute for Health Research, University of Notre Dame, Fremantle, WA, 6959, Australia; ^6^ Department of Medical Oncology, Sir Charles Gairdner Hospital, Nedlands, WA, 6009, Australia

**Keywords:** rectal cancer, regulatory T cells, radiotherapy, chemotherapy, treatment response

## Abstract

Neoadjuvant (preoperative) chemoradiotherapy (CRT) decreases the risk of rectal cancer recurrence and reduces tumour volume prior to surgery. However, response to CRT varies considerably between individuals and factors associated with response are poorly understood. Foxp3^+^ regulatory T cells (Tregs) inhibit anti-tumour immunity and may limit any response to chemotherapy and radiotherapy. We have previously reported that a low density of Tregs in the tumour stroma following neoadjuvant CRT for rectal cancer is associated with improved tumour regression. Here we have examined the association between Treg density in pre-treatment diagnostic biopsy specimens and treatment response, in this same patient cohort. We aimed to determine whether pre-treatment tumour-infiltrating Treg density predicts subsequent response to neoadjuvant CRT. Foxp3^+^, CD8^+^ and CD3^+^ cell densities in biopsy samples from 106 patients were assessed by standard immunohistochemistry (IHC) and evaluated for their association with tumour regression grade and survival. We found no association between the density of any T cell subset pre-treatment and clinical outcome, indicating that tumour-infiltrating Treg density does not predict response to neoadjuvant CRT in rectal cancer. Taken together with the findings of the previous study, these data suggest that in the context of neoadjuvant CRT for rectal cancer, the impact of chemotherapy and/or radiotherapy on anti-tumour immunity may be more important than the state of the pre-existing local immune response.

## INTRODUCTION

Colorectal cancer is the third most common cancer worldwide, representing almost 10% of all cancer diagnoses [1]. Rectal cancer accounts for around one third of colorectal cancer cases. Standard treatment for locally advanced rectal cancer consists of neoadjuvant radiotherapy with concurrent 5-fluorouracil (5-FU)-based chemotherapy (neoadjuvant CRT), followed by surgery. Neoadjuvant CRT reduces tumour volume and decreases the risk of local recurrence [2, 3]. However, response (degree of regression) varies considerably between individuals. While approximately 20% of patients have a pathologic complete response (defined as no viable tumour cells in the surgical resection specimen), around 60% have a partial or intermediate response, and approximately 20% experience little to no regression [4–6]. The benefit of CRT for this latter patient group is therefore less clear and factors associated with response are not well understood. Importantly, the extent of tumour regression following CRT is associated with improved long-term outcome, even in the context of potentially curative surgery [7–9].

It is well established that the immune system plays an important role in cancer control and treatment response [10, 11]. Infiltration of tumours by T cells is associated with improved prognosis in many cancers [12–15] and may even be more prognostic than histological TNM stage for patients with stage I-III colorectal cancer [16]. In rectal cancer patients treated with surgery alone, a higher density of total and CD8^+^ T cells in the central tumour and invasive margin is associated with improved disease-free and overall survival [17], and in neoadjuvantly-treated rectal cancer, a greater pre-treatment CD8^+^ T cell infiltrate predicts good response to CRT [17–19]. In contrast, tumour infiltration by CD4^+^ regulatory T cells (Tregs), which constitutively express the transcription factor forkhead box protein 3 (Foxp3) and negatively regulate immune responses, is often associated with poorer clinical outcome [20–23].

Both chemotherapy and radiotherapy can enhance anti-tumour immunity through a range of different mechanisms, including selective depletion of Tregs [24–28]. We recently reported that, in a cohort of 128 patients who underwent neoadjuvant CRT and surgery for rectal cancer at our institution, the presence of fewer Tregs in the stroma of surgical resection specimens (post-CRT) was strongly associated with improved tumour regression [29]. Patients with a low stromal Treg density were over five times more likely to have a complete pathological response (pCR) after adjustment for clinical factors. While this may represent selective Treg depletion in these patients, an alternate hypothesis is that fewer Tregs in the local tumour environment prior to treatment may allow for effective chemoradiotherapy-induced antitumour immunity.

Here we present an evaluation of Foxp3^+^, CD8^+^ and CD3^+^ cell density in pre-treatment diagnostic biopsy samples from this same cohort, with the aim of determining whether pre-CRT T cell density can predict subsequent treatment response.

## RESULTS

Diagnostic biopsy samples with sufficient material remaining for immunohistochemistry (IHC) analysis were obtained for 113 patients (88% of our originally reported cohort [29]). Eight cases were stained for Foxp3 and CD8 only. Six cases were judged to be of inadequate staining quality (detached/folded tissue or high non-specific background DAB staining) and were subsequently excluded (five cases for CD3 only and one case for all stains). Three cases were found to have no invasive cancer remaining in the sample and were excluded from all analyses. To minimise sampling bias, samples with a total analysis area (sum of all fragments) <1mm^2^ (a further three patients) were also excluded. Of the remaining 106 cases, median total tissue area analysed per sample was 6 mm^2^. Moderate, but significant concordance in T cell subset densities between individual biopsy fragments selected for analysis was observed (Supplementary Figure 1B).

Pre-treatment characteristics, treatment response and infiltrating T cell densities for the 106 patients included in the analyses are shown in Table 1. The majority of patients were male and the mean age at time of surgery was 62 years. Most (83%) patients were clinical T stage 3, and 79% were assessed as having lymph node involvement on pre-treatment imaging. According to the Dworak grading system [30], 21 patients (20%) had a complete response to CRT (Dworak grade 4), 38 patients (36%) a good response (Dworak grade 3), 40 (38%) an intermediate response (Dworak grade 2) and 7 patients (6%) were classed as Dworak 1 poor responders.

**Table 1:** Patient characteristics

	n = 106
Age, mean (sd)	62 (12.3)
Gender, n (%)	
male	77 (73)
female	29 (27)
Distance from anal verge (cm), mean (sd)	7 (3.6)
Weeks between end CRT and surgery, median (IQR)	7 (6, 8)
Pre-treatment clinical T stage, n (%)	
T2	7 (7)
T3	86 (83)
T4	11 (11)
NR	2 (2)
Pre-treatment clinical N stage, n (%)	
N0	22 (21)
N1-2	82 (79)
NR	2 (2)
Pre-treatment clinical M stage, n (%)	
M0	95 (90)
M1-2	10 (9)
NR	1 (1)
Dworak grade, n (%)	
4 (pCR)	21 (20)
3	38 (36)
2	40 (38)
1	7 (6)
Foxp3^+^ cell density (cells / mm^2^), median (range)	427 (42, 1562)
CD8^+^ cell density (cells / mm^2^), median (range)	373 (29, 1814)
CD3^+^ cell density (cells / mm^2^), median (range)	1104 (108, 2514)*

Abbreviations: IQR = interquartile range; NR = not recorded; pCR = pathologic complete response

* n = 93 (8 cases were stained for Foxp3 and CD8 only; a further 5 cases were excluded due to poor staining/tissue quality)

**Figure 1:**
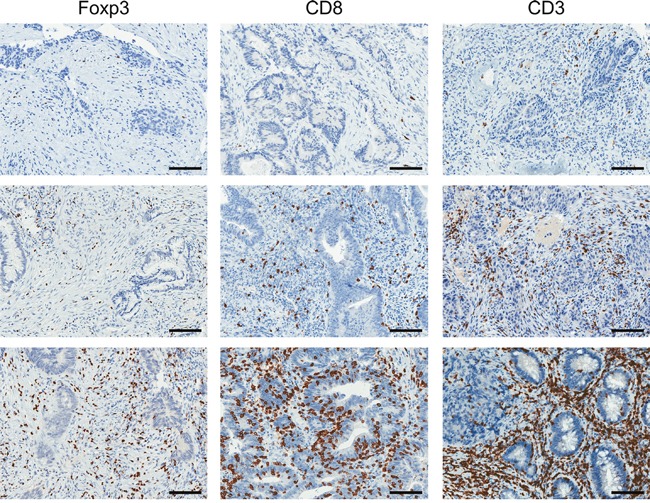
Identification of T cell subsets in diagnostic biopsy samples by immunohistochemistry Representative images (from different patients) demonstrating low (top row), moderate (middle row) and high (bottom row) densities of Foxp3^+^, CD8^+^ and CD3^+^ cells. Scale bar, 100μm.

**Figure 2:**
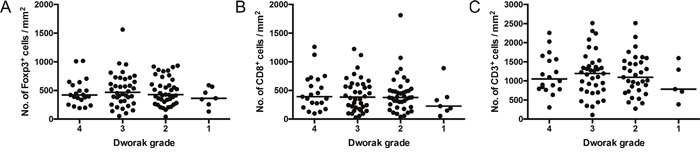
Pre-CRT T cell density does not predict treatment response Foxp3^+^ **A**., CD8^+^ **B**. and CD3^+^ **C**. cell densities by tumour regression (Dworak) grade. P = NS; general linear model. Dots represent individual patients. Line at median.

**Figure 3:**
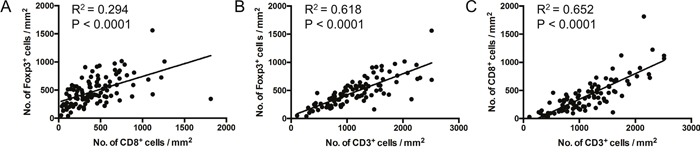
Correlation between T cell subset densities Linear regression analyses. Dots represent individual patients. P < 0.05 indicates significant correlation.

We observed considerable variation in infiltrating T cell densities between patients (Table 1, Figure 1 and Supplementary Figure 1). However, analysis of T cell subset density by Dworak grade revealed no association between pre-treatment Foxp3^+^, CD8^+^ or CD3^+^ cell densities and response to CRT (Figure 2). There was no significant difference in T cell subset densities when Dworak 3-4 ‘good’ responders were compared to Dworak 1-2 poor/intermediate responders, or when Dworak 4 complete responders were compared to all other groups (data not shown). There was a strong positive correlation between the density of Foxp3^+^ cells and CD8^+^ cells within individual samples (Figure 3A). As expected, densities of CD8^+^ and CD3^+^ cells, and of Foxp3^+^ and CD3^+^ cells were also strongly correlated (Figure 3B and 3C). Ratios of CD8^+^ to CD3^+^ T cells and Foxp3^+^ to CD8^+^ T cells were also assessed for correlation with tumour regression grade, but no association was found (data not shown).

We next investigated the relationship between T cell subset densities and pre-treatment clinical and demographic factors (Table 2). Gender was the only variable significantly associated with infiltrating T cells, with a higher density of Foxp3^+^, and CD8^+^ cells in biopsies from female compared to male patients.

**Table 2:** T cell subset densities according to pre-treatment clinical and demographic factors

		Foxp3^+^ cell density			CD8^+^ cell density			CD3^+^ cell density		
		Lown (%)	Highn (%)	P	Lown (%)	Highn (%)	P	Lown (%)	Highn (%)	P
Age	≥ 65	24 (47)	27 (53)	0.560	24 (47)	27 (53)	0.560	20 (45)	24 (55)	0.353
	< 65	29 (53)	26 (47)		29 (53)	26 (47)		27 (55)	22 (45)	
Gender	Male	44 (57)	33 (43)	**0.017**	44 (57)	33 (43)	**0.017**	36 (55)	30 (45)	0.227
	Female	9 (31)	20 (69)		9 (31)	20 (69)		11 (41)	16 (59)	
Distance from anal verge	≥ 7cm	28 (49)	29 (51)	0.846	28 (49)	29 (51)	0.846	25 (50)	25 (50)	0.911
	< 7cm	25 (51)	24 (49)		25 (51)	24 (49)		22 (51)	21 (49)	
Clinical T stage	T2	4 (57)	3 (43)	0.246	2 (29)	5 (71)	0.395	1 (17)	5 (83)	0.207
	T3	40 (47)	46 (53)		43 (50)	43 (50)		39(52)	36(48)	
	T4	8 (73)	3 (27)		7 (64)	4 (36)		6(60)	4(40)	
Clinical N stage	N0	14 (64)	8 (36)	0.150	12 (55)	10(45)	0.631	10 (48)	11 (52)	0.759
	N1-2	38 (45)	44 (54)		40 (49)	42 (51)		36 (51)	34 (49)	
Clinical M stage	M0	47 (49)	48 (51)	0.742	47 (49)	48 (51)	0.975	41 (49)	43 (51)	0.714
	M1-2	6 (60)	4 (40)		5 (50)	5 (50)		5 (63)	3 (37)	

Bold text denotes significant associations (P < 0.05)

**Figure 4:**
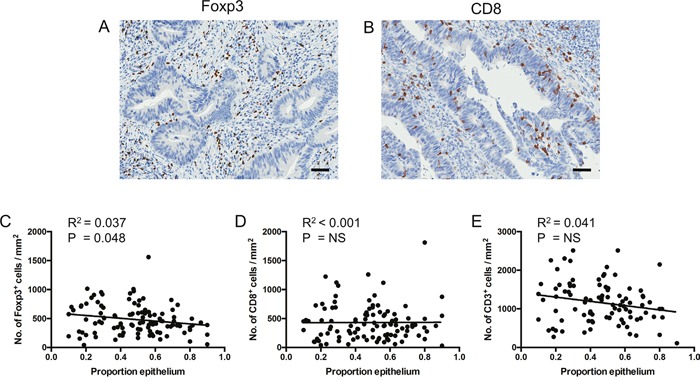
Foxp3+ cell density is inversely proportional to epithelial content **A** and **B**. Representative images (from different patients) demonstrating Foxp3^+^ cells located in the stroma (A) and CD8^+^ cells infiltrating the epithelium (B). Scale bar, 50μm. **C** to **E**. Linear regression analyses of T cell subset density by estimated epithelial tissue content across fragments. Dots represent individual patients. Data represent totals across all fragments analysed. NS = not significant.

Visual inspection of IHC images suggested that Foxp3^+^ T cells were located primarily in the stroma, rarely infiltrating the epithelial tissue (Figure 4A). Linear regression analysis confirmed an inversely proportional relationship between Foxp3^+^ T cell density and estimated epithelial content (Figure 4C). However, adjusting for epithelial content did not reveal any association between Foxp3^+^ T cell density and Dworak grade (data not shown).

**Figure 5:**
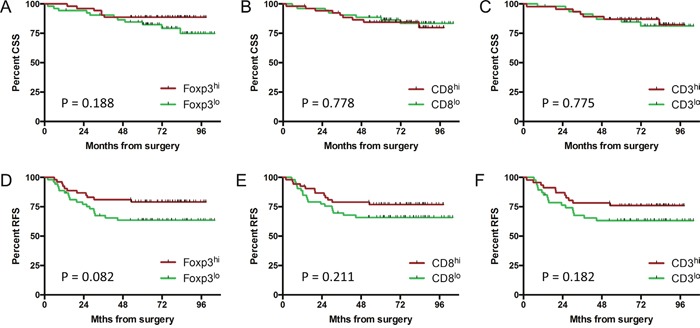
Pre-CRT T cell density is not associated with long-term survival Kaplan-Meier estimates for cancer-specific **A-C**. and recurrence-free survival **D-F**. by T cell subset density. Groups were split at the median value. P = NS; log-rank test.

Median follow up duration from surgery for this patient group was 78 months, with 5-year cancer-specific survival (CSS) and recurrence-free survival (RFS) of 87% and 83% respectively. Kaplan-Meier survival analysis demonstrated that pre-treatment T cell density had no prognostic value in this patient group (Figure 5).

**Figure 6:**
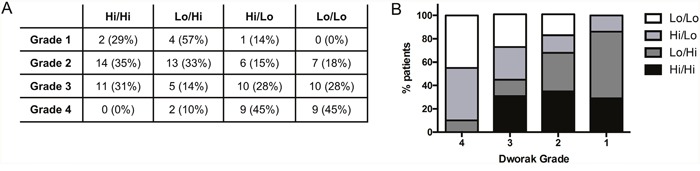
Foxp3+ cell density pre and post-CRT Patients with available data at both biopsy and resection (n = 103) were divided into four groups based on the density of Foxp3^+^ cells in the pre-treatment biopsy sample and in the stroma at resection (high *versus* low, split at the median value). The frequency **A**. and percentage (A) and **B**. of patients in each group by Dworak grade.

These data, together with our previous finding that a low stromal Foxp3^+^ Treg density post-CRT was associated with greater tumour regression in this cohort [29], suggest that the impact of CRT on anti-tumour immunity may be more important than the state of the pre-existing local immune response. We divided the cohort into four groups according to Treg density in the pre-treatment biopsy samples and in the post-treatment stroma (high/high, low/high, high/low, and low/low). While only 10% of patients who achieved a Dworak 4 pCR had a stromal Treg density higher than the median value post-CRT, patients in this group were equally likely to have a high or low Treg density pre-CRT (Figure 6). Similarly, while only 14% of Dworak 1 poor responders had a low stromal Treg density post CRT, the percentage of patients in this group with a high *vs* low pre-CRT Treg density was 43% *vs* 57% respectively.

## DISCUSSION

In this study, we investigated the relationship between T cell subset density in pre-treatment diagnostic biopsies and response to neoadjuvant CRT in 106 patients with locally advanced rectal cancer. We hypothesised that an existing local anti-tumour immune response dominated by CD8^+^ cells rather than Tregs would ‘set the scene’ for effective CRT-induced anti-tumour immunity, and therefore better treatment response. Despite considerable inter-patient variation in T cell subset densities, we found no association between Foxp3^+^, CD8^+^ or CD3^+^ cell density (or ratios of T cell subsets) in pre-treatment biopsies and tumour regression grade or long-term survival.

T cell subset densities did not differ according to clinical tumour or nodal stage, or the presence / absence of distant metastases as determined by pre-treatment imaging. The only clinical or demographic variable significantly associated with T cell subset density was gender, with females having a more pronounced infiltrate of Foxp3^+^and CD8^+^ cells. While an interesting observation, this finding is unlikely to be clinically important since there was no gender-specific difference in CRT response or survival in this, or in previously studied cohorts [18, 19, 29, 31] (data not shown).

We were not able to replicate the finding from previous similar studies that a high density of CD8^+^ T cells prior to neoadjuvant treatment is associated with good response [17–19]. A key difference between these studies and ours is that we assessed T cell subset densities objectively across entire tissue fragments using image analysis software (Supplementary Figure 2), whereas the studies by Teng *et al* and Yasuda *et al* determined T cell subset densities by visual scoring of randomly selected high power fields [18, 19]. Anitei *et al* used image analysis software, but calculated cell density based on the average of the three most infiltrated areas [17]. Also, over 99% of our patients received concurrent chemotherapy, compared to 76% in the study reported by Anitei *et al* (24% receiving radiotherapy alone) [17]. Another possible reason for discordance with these previous studies could be the different systems used for assessing response to CRT. While Teng *et al* also used the Dworak system, Anitei *et al* used a modified three-tier grading system based on tumour-fibrosis ratio [32] and Yasuda *et al* evaluated response according to the Japanese Classification of Colorectal Carcinoma [33]. Importantly, there is inherent imprecision in current systems used to assess tumour regression following neoadjuvant therapy due to the subjective nature of terms used (such as ‘few tumour cells’, ‘easy to find’ and ‘significant fibrosis’), leaving them open to individual interpretation. This subjectivity could also explain the relatively low proportion of patients classed as Dworak 1 poor responders in our cohort, compared to some previous studies [4–6].

In another recent study, Shinto *et al* found a high CD8/Foxp3 ratio pre-CRT to be predictive of improved tumour regression [34]. However, patients in that study received short-course CRT (20 Gy given over 5 days with concurrent uracil over 7 days) and surgery 30 days later, rather than long-course CRT (50.4 Gy over 5 weeks with concurrent 5-FU-based chemotherapy) as received by patients in our cohort. Complete response is less common after short-course CRT and tumour regression can continue for up to 12 weeks following long-course CRT [35], thus these reported results may not be relevant to long-course CRT.

In the context of long-course CRT, the impact of chemotherapy and/or radiotherapy on anti-tumour immunity may be more important than the state of the pre-existing local immune response. In this same patient cohort, we recently demonstrated that a low density of Foxp3^+^ Tregs within the tumour stroma following neoadjuvant CRT was strongly associated with pCR [29]. Indeed, when both pre- and post-treatment Treg density was taken into account in the current study, while 90% of the Dworak 4 complete responders had a post-CRT stromal Treg density below the median value, the number of patients with a high *versus* low pre-CRT Treg density in this group was equal.

Tregs undergo rapid turnover relative to other T cell subsets and are selectively depleted by several chemotherapy drugs, including 5-FU [28, 36, 37]. It is possible that selective depletion of Tregs during CRT in some patients may create a ‘window of opportunity’ for enhanced anti-tumour immunity, contributing to tumour regression. However, we cannot rule out that an increased presence of Tregs surrounding poor responding tumours post-CRT simply reflects an immune response to residual tumour. The anti-tumour immune response is also inherently complex. It may be that the functional status of T cells within the tumour microenvironment prior to CRT, and/or their interaction with other cell types, is more important than simply the density of the major T cell subsets. We plan to address this in a second similar cohort.

Although tumour-infiltrating Foxp3^+^ cells are associated with poor prognosis in most solid cancers [20–23], their presence in colorectal cancer has proven to be a good prognostic factor in several independent studies [38–40]. Treg expansion in this context may simply represent an indirect measure of anti-tumour immunity (in response to CD8^+^ T cell activation); the densities of these subsets do often correlate [39, 41, 42]. It has also been proposed that Tregs limit the pro-tumour effects of Th17-mediated inflammation, driven by the presence of gut bacteria [43, 44]. Alternatively, the positive impact of Foxp3^+^ T cells in the gut may be due to a subset of non-suppressive effector/memory T cells that express Foxp3 at lower levels. These are found in higher numbers in colorectal tumours than in other cancers, but cannot be distinguished from suppressive Tregs using standard immunohistochemical methods [45]. A weakness of the current study is that, as we too were using standard IHC, our staining could not differentiate Tregs from other Foxp3-expressing cell subsets.

The study was powered to detect a 15% change in response rate on the continuum between lowest and highest cell subset density with 90% power at a 5% significance level (100 observations required). We cannot exclude the possibility that more subtle associations with response could be revealed with a larger cohort.

In conclusion, we show that Foxp3^+^ cell density in the tumour microenvironment prior to CRT did not predict tumour regression in locally advanced rectal cancer. Further work in this area is required to investigate the potential role of the immune system in mediating response to CRT.

## MATERIALS AND METHODS

### Patients

Consecutive patients with rectal adenocarcinoma treated between 2006 and 2010 with long-course neoadjuvant 5-fluorouracil (5-FU)-based CRT and surgery were identified from our prospectively maintained institutional database. Clinical data collection, treatment and follow up have been described previously [29]. Dworak tumour regression grade [30] was assigned by the gastrointestinal pathologists at the time of routine pathology reporting, and subsequently verified by a single pathologist.

Grade 4: no tumour cells, only fibrotic mass (pCR)

Grade 3: very few (difficult to find microscopically) tumour cells in fibrotic tissue

Grade 2: dominantly fibrotic changes with few tumour cells or groups (easy to find)

Grade 1: dominant tumour mass with obvious fibrosis and/or vasculopathy

Grade 0: no regression

### Immunohistochemistry

Archived pre-treatment diagnostic biopsy material (formalin-fixed paraffin-embedded (FFPE) tissue) was obtained. Where there was sufficient material remaining, 4μm sections were cut and stained for Foxp3, CD8 and CD3 by standard immunohistochemistry (IHC), as previously described [29]. Slides were scanned at 40x magnification using a high-resolution digital scanner (Aperio Scanscope XT; Leica Biosystems, North Ryde, NSW, Australia). A specialist gastrointestinal histopathologist (CH) reviewed corresponding H&E slides for all cases and selected up to three fragments containing invasive cancer for digital image analysis (Supplementary Figure 2A). For each fragment, the proportion of epithelial tissue was also estimated to the nearest 10% (or 5% if <10%).

### Digital image analysis

Evaluation of Foxp3^+^, CD8^+^ and CD3^+^ T cell density (cells / mm^2^ tissue) was performed using StrataQuest version 5 (TissueGnostics, Taborstraße, Vienna, Austria), as shown in Supplementary Figure 2B-2D. All images were evaluated for staining quality before digital analysis. Data represent the total density across all fragments (total number of positive cells / total area).

To assess possible differences in automated compared to visual cell counts, ten small regions of interest (ROI; mean area 0.88 mm^2^) were selected on the digital images of Foxp3-stained biopsies for comparison of counting methods. Three independent observers assessed Foxp3^+^ cell count and the regions were then subjected to automated image analysis. A high level of correlation was observed between the visual and automated methods, as well as between independent observers (Supplementary Figure 3).

### Statistical analysis

Differences in T cell density between response groups, and associations between clinical/demographic variables and T cell density, were assessed using general linear models. Survival analyses were performed using the Kaplan-Meier method with curves compared using the log-rank test. Associations between clinical variables and T cell densities were assessed using the chi-square test, or Fisher's exact test where there were fewer than 5 observations in any group. To compare high *vs* low T cell subset densities, the group was split at the median value. Differences and associations were considered statistically significant where P < 0.05. Median follow up time was calculated using the reverse Kaplan-Meier method. Analyses were performed using SAS version 9.4 (SAS Institute Inc, Cary, NC, USA) and GraphPad Prism version 6.0 (GraphPad software Inc, San Diego, CA, USA).

### Ethical statement

The study was approved by the St John of God Health Care Human Research Ethics Committee and conducted in accordance with the Declaration of Helsinki. All patients gave written informed consent for their biospecimens and health information to be used for research purposes.

## SUPPLEMENTARY MATERIALS FIGURES



## Abbreviations

5-FU: 5-fluorouracil
CRT: chemoradiotherapy
FFPE: formalin-fixed paraffin-embedded
IHC: immunohistochemistry
NS: not significant
pCR: pathological complete response
TILs: tumour-infiltrating lymphocytes
Tregs: regulatory T cells
