# Supplementary material for: Tumour-infiltrating regulatory T cell density before neoadjuvant chemoradiotherapy for rectal cancer does not predict treatment response
Source: Oncotarget. 2017 Feb 3;8(12):19803–13. doi: 10.18632/oncotarget.15048 (PMC5386723; doi:10.18632/oncotarget.15048)
Supplement: Supplementary file 1 [file oncotarget-08-19803-s001.pdf]

# Tumour-infiltrating regulatory T cell density before neoadjuvant chemoradiotherapy for rectal cancer does not predict treatment response

## SUPPLEMENTARY FIGURES

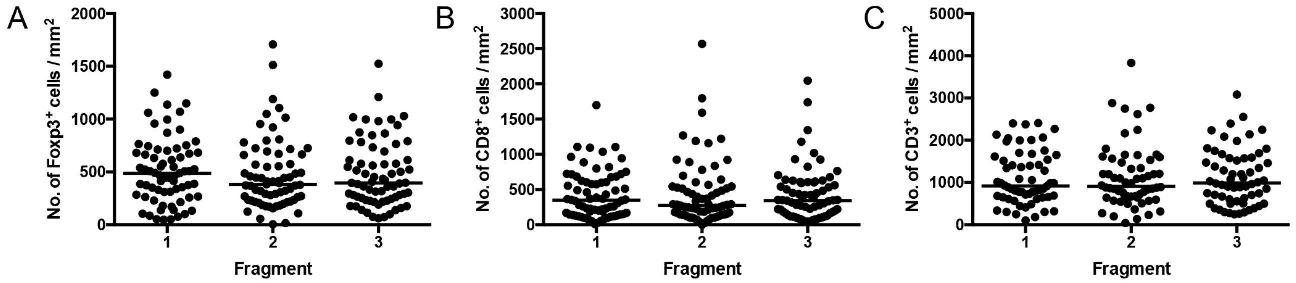


D

| | Kendall's W | P value |
|---|---|---|
| Foxp3 | 0.58 | 0.0002 |
| CD8 | 0.68 | <.0001 |
| CD3 | 0.61 | 0.0001 |

**Supplementary Figure 1: T cell subset densities across biopsy fragments. A-C.** Foxp3$^+$, CD8$^+$ and CD3$^+$ cell densities by fragment. Dots represent individual fragments, line at median. **D.** Concordance in T cell densities between fragments for all patients with three fragments available for analysis (n = 69, 70 and 60 for Foxp3, CD8 and CD3 respectively). Performed using STATA/SE version 14 (StataCorp LP, College Station, Texas, USA). $P < 0.05$ indicates significant concordance.

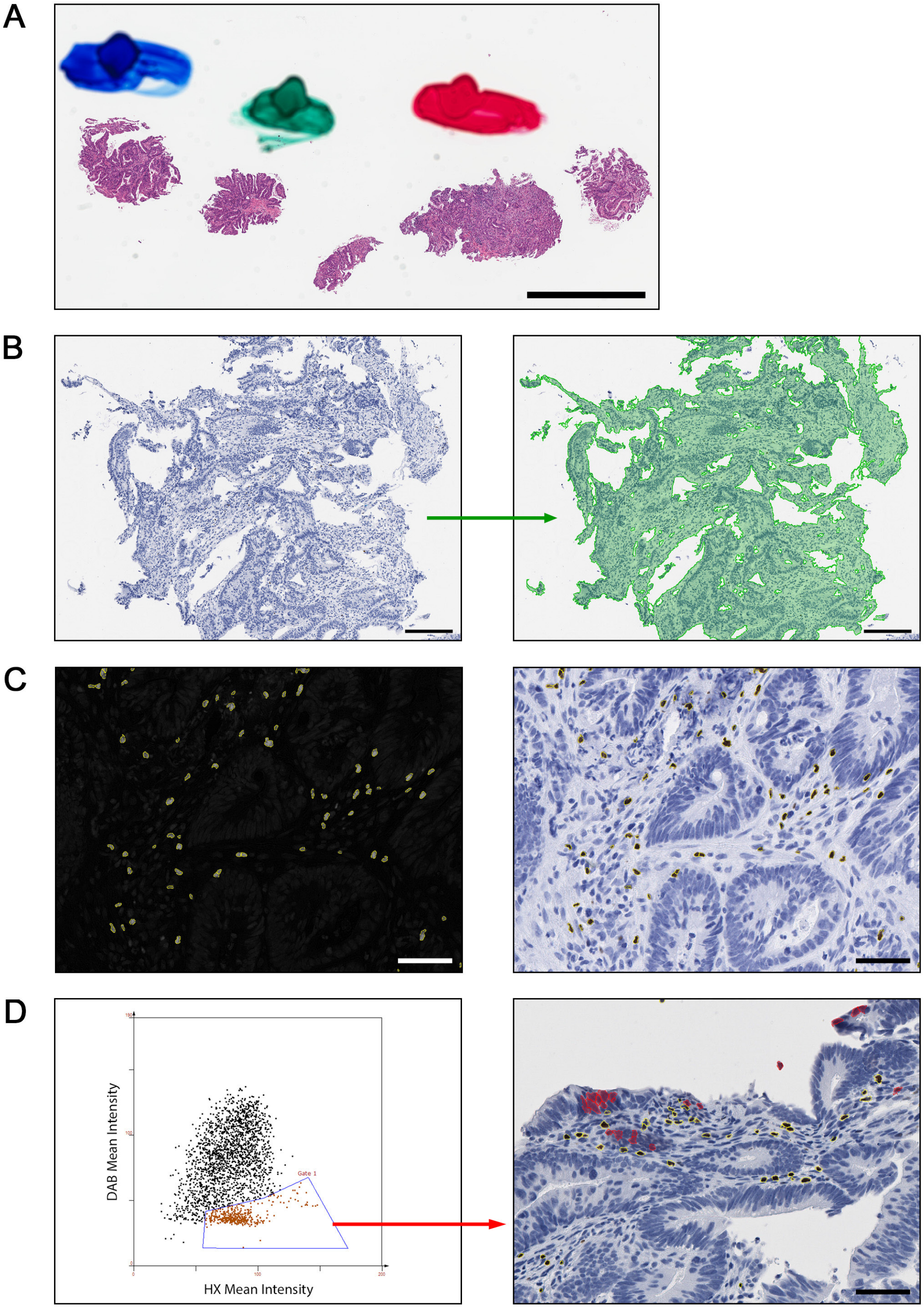


**Supplementary Figure 2: Analysis workflow. A.** Representative H&E section from a tumour biopsy with three fragments containing invasive cancer marked for analysis (red, blue and green = fragments 1, 2 and 3 respectively). Scale bar, 2 mm. **B-D.** StrataQuest image analysis for Foxp3 showing (B) tissue mask overlay to define analysis area. Scale bar, 200 µm. (C) Colour deconvolution of DAB stain and nuclear segmentation of DAB$^{+}$ cells (L), overlaid onto original image (R). Scale bars, 50 µm. (D) Identification of haematoxylin strong, DAB weak cells (false positives) (L). Backward connection to original image with nuclear segmentation. Gated cells shown in red (R). Scale bar, 50 µm. Foxp3$^{+}$ cell density was calculated as (# DAB$^{+}$ cells - # false positives) / analysis area.

A

| | Visual Counts | | | | Automated count |
|---|---|---|---|---|---|
| | Observer 1 | Observer 2 | Observer 3 | Median | (DAB Nuclear Segmentation) |
| ROI 02 | 17 | 20 | 18 | 18 | 20 |
| ROI 04 | 25 | 29 | 28 | 28 | 26 |
| ROI 01 | 30 | 34 | 33 | 33 | 34 |
| ROI 03 | 44 | 52 | 42 | 44 | 48 |
| ROI 06 | 49 | 52 | 46 | 49 | 54 |
| ROI 05 | 43 | 56 | 51 | 51 | 55 |
| ROI 09 | 53 | 51 | 49 | 51 | 53 |
| ROI 10 | 59 | 68 | 55 | 59 | 63 |
| ROI 08 | 228 | 220 | 212 | 220 | 236 |
| ROI 07 | 256 | 325 | 296 | 296 | 433 |

B

| | Observer 1 | Observer 2 | Observer 3 | Median | Automated |
|---|---|---|---|---|---|
| Observer 1 | | 0.888** | 0.927*** | 0.948*** | 0.915*** |
| Observer 2 | 0.888** | | 0.960*** | 0.945*** | 0.979*** |
| Observer 3 | 0.927*** | 0.960*** | | 0.997*** | 0.988*** |
| Median | 0.948*** | 0.945*** | 0.997*** | | 0.979*** |
| Automated | 0.915** | 0.979*** | 0.988*** | 0.979*** | |

**Supplementary Figure 3: Visual versus automated Foxp3$^+$ cell counts.** Ten regions of interest (ROI; mean area 0.88 mm$^2$) were selected on the digital images from five different patients. Regions were selected to represent a range in Foxp3$^+$ cell densities, as determined by eye. The number of Foxp3$^+$ cells was counted by three independent observers and the regions were then subjected to automated image analysis using StrataQuest version 5 (TissueGnostics, Taborstraße, Vienna, Austria), using our optimised analysis profile based on DAB nuclear staining segmentation. **A.** Number of Foxp3$^+$ cells per ROI, ordered by increasing Foxp3$^+$ cell density according to the median visual count. **B.** Spearman correlation coefficient for each pair-wise comparison; ** $p < 0.01$, *** $p < 0.001$.
